# Patient and Clinician Perspectives of a Standardized Question About Firearm Access to Support Suicide Prevention: A Qualitative Study

**DOI:** 10.1001/jamahealthforum.2022.4252

**Published:** 2022-11-23

**Authors:** Julie E. Richards, Elena S. Kuo, Ursula Whiteside, Lisa Shulman, Marian E. Betz, Rebecca Parrish, Jennifer M. Boggs, Ali Rowhani-Rahbar, Gregory E. Simon

**Affiliations:** 1 Kaiser Permanente Washington Health Research Institute, Seattle; 2 Department of Health Systems & Population Health, School of Public Health, University of Washington, Seattle; 3 Psychiatry and Behavioral Sciences, University of Washington, Seattle; 4 NowMattersNow.org, Seattle, Washington; 5 Department of Emergency Medicine, University of Colorado School of Medicine, Aurora; 6 Kaiser Permanente Washington Department of Mental Health & Wellness, Seattle; 7 Kaiser Permanente Colorado Institute for Health Research, Aurora; 8 Department of Epidemiology, School of Public Health, University of Washington, Seattle; 9 Harborview Injury Prevention & Research Center, Seattle, Washington

## Abstract

**Importance:**

US residents report broad access to firearms, which are the most common means of suicide death in the US. Standardized firearm access questions during routine health care encounters are uncommon despite potential benefits for suicide prevention.

**Objective:**

To explore patient and clinician experiences with a standard question about firearm access on a self-administered mental health questionnaire routinely used prior to primary care and mental health specialty encounters.

**Design, Setting, and Participants:**

Qualitative semistructured interviews were conducted from November 18, 2019, to October 8, 2020, at Kaiser Permanente Washington, a large integrated care delivery system and insurance provider. Electronic health record data identified adult patients with a documented mental health diagnosis who had received a standard question about firearm access (“Do you have access to guns? yes/no”) within the prior 2 weeks. A stratified sampling distribution selected 30% who answered “yes,” 30% who answered “no,” and 40% who left the question blank. Two groups of clinicians responsible for safety planning with patients at risk of suicide were also sampled: (1) licensed clinical social workers (LICSWs) in primary and urgent care settings and (2) consulting nurses (RNs).

**Main Outcomes and Measures:**

Participants completed semistructured telephone interviews, which were recorded and transcribed. Directive (deductive) and conventional (inductive) content analyses were used to apply knowledge from prior research and describe new information. Thematic analysis was used to organize key content, and triangulation was used to describe the intersections between patient and clinician perspectives.

**Results:**

Thirty-six patients were interviewed (of 76 sampled; mean [SD] age, 47.3 [17.9] years; 19 [53%] were male; 27 [75%] were White; 3 [8%] were Black; and 1 [3%] was Latinx or Hispanic. Sixteen participants had reported firearm access and 15 had reported thoughts of self-harm on the questionnaire used for sampling. Thirty clinicians were interviewed (of 51 sampled) (mean [SD] age, 44.3 [12.1] years; 24 [80%] were female; 18 [60%] were White; 5 [17%] were Asian or Pacific Islander; and 4 [13%] were Latinx or Hispanic) including 25 LICSWs and 5 RNs. Key organizing themes included perceived value of standardized questions about firearm access, challenges of asking and answering, and considerations for practice improvement. Clinician interview themes largely converged and/or complemented patient interviews.

**Conclusions and Relevance:**

In this qualitative study using semistructured interviews with patients and clinicians, a standardized question about firearm access was found to encourage dialogue about firearm access. Respondents underscored the importance of nonjudgmental acknowledgment of patients’ reasons for firearm access as key to patient-centered practice improvement.

## Key Points

**Question:**

How did patients experience answering and clinicians experience using a standard question about firearm access on a routine mental health questionnaire?

**Findings:**

In this qualitative study, a standardized question about firearm access was asked during semistructured interviews with 36 patients, including firearm owners and people who were experiencing suicidal thoughts, and 30 clinicians, including clinical social workers and nurses. Participants described why firearm access questions are important, but can be challenging to answer and discuss.

**Meaning:**

These findings suggest that firearm access questions can be used to normalize and support patient-centered dialogue about when and how to limit access to firearms to reduce risk of suicide.

## Teaser

This qualitative study samples patients with recent experiences answering a firearm question in a large regional health care system and clinicians responsible for engaging patients at risk of suicide in risk mitigation and follow-up care to identify multidimensional practice barriers and facilitators for improving the routine use of standard questions about firearms during mental health care encounters.

## Introduction

Suicide accounts for more than half of firearm deaths in the US,^1^ and 85% to 95% of people who attempt suicide by firearm do not survive.^2^ Nationally, approximately a third of US residents report owning firearms,^3^ 42% report household firearms,^4^ 3% acquired firearms during the COVID-19 pandemic,^5^ and ownership of multiple firearms and firearm types (eg, handguns, long guns) is common.^6^ People with firearm access, particularly if stored loaded and unlocked,^7,8^ have increased suicide risk.^9,10^ Research suggests approximately half of adults who die by suicide see a health care provider in the prior month and nearly all in the prior year.^11,12^ Therefore, risk identification using standard firearm access questions, such as those used for suicidal thoughts (now recommended by the Joint Commission),^13,14^ may facilitate engaging patients in interventions such as collaborative safety planning^15^ and reducing access to firearms during periods of increased suicide risk.^16,17,18^

Major medical associations recommend that clinicians counsel patients at risk of suicide to limit firearm access.^19^ However, standardized population-based questions regarding firearm access are uncommon; typically organizations rely on clinician discretion to query patients.^20^ This practice undoubtedly results in incomplete information,^21,22^ as clinicians often have limited time^23,24^ or knowledge about querying firearm access.^25^ Moreover, gun control is polarizing in the US,^26^ and clinicians may worry about harming relationships and therapeutic alliances with patients.^27^ No federal law or statute prohibits asking about firearms for prevention,^27^ but clinicians have expressed fears about regulations.^28,29^ These factors have likely contributed to the lack of available evidence to guide clinical recommendations regarding patient-reported firearm access practices.^20^ Understanding patient and clinician experiences with standard firearm access questions is critical for informing practice implementation and improvement. Prior qualitative research has highlighted that patient concerns about losing firearm access pose a barrier to assessment.^30,31,32,33^ Moreover, a growing body of qualitative research supports transparency, context, nonjudgment, and trust as key to overcoming patient hesitancy around reporting firearm access.^31,32,33,34,35,36,37,38,39^ However, to our knowledge no studies to date have explored actual experiences with standardized firearm access questions during routine health care encounters, perhaps due to the rarity of this clinical practice and the lack of opportunities to research this topic.

Therefore, this qualitative study aimed to purposefully sample (1) patients with recent experiences answering a firearm question on a routine mental health monitoring questionnaire implemented by a large regional health care system^40^ and (2) clinicians responsible for engaging patients at risk of suicide in risk mitigation and follow-up care. Semistructured interviews were designed to elicit multidimensional practice barriers and facilitators for purposes of informing and improving the routine use of standard questions about firearms during mental health care encounters.

## Methods

### Setting

Participants were recruited from Kaiser Permanente Washington (KPWA), a large integrated care delivery system and insurance provider serving approximately 700 000 Washington State residents. Kaiser Permanente Washington added the question “Do you have access to guns? yes/no” to a mental health monitoring questionnaire to support suicide prevention practices.^16^ The questionnaire was implemented across all mental health and primary care clinics between 2016 and 2018.^40^ Patients with current mental health or substance use disorder diagnoses typically completed the questionnaire on paper before appointments, and staff entered responses into the electronic health record (EHR) during appointments. Data collection and qualitative interview activities were approved by the KPWA institutional review board and followed the Consolidated Criteria for Reporting Qualitative Research (COREQ) reporting guideline.^41^

### Patient Recruitment

The EHR data identified adult patients (aged ≥18 years) with a documented mental health diagnosis who had received a standard question about firearm access (“Do you have access to guns? yes/no”), within the prior 2 weeks. A stratified sampling distribution was used to recruit patients who (1) reported access, (2) reported no access, and (3) left the question blank; patients were purposefully sampled from each group who also reported having self-harm thoughts (eMethods 1 in the Supplement).^42^ Patients were mailed invitations, including an information sheet with instructions for opting out. Interviewers attempted to call invited patients within 2 weeks of invitation and recruitment continued in waves until thematic saturation was reached.^43^

### Clinician Recruitment

Two groups of clinicians were recruited: (1) licensed clinical social workers (LICSWs) (n = 43) responsible for collaborative safety planning^15^ in primary and urgent care settings and (2) consulting nurses (RNs) (n = 8) responsible for connecting patients reporting suicidality after business hours to follow-up care by telephone. Clinicians received up to 3 email invitations, including an information sheet with instructions for opting out.

### Telephone Interviews

All participants provided oral consent for participation in semistructured interviews, including consent to publish direct quotations. In addition, patients consented to use of their EHRs to describe participant characteristics. Three female interviewers, including 2 doctoral-level public health researchers (J.R. and E.K.) and 1 masters-level social worker (L.S.), conducted semistructured telephone interviews (~20 minutes). Interview guides were developed from prior qualitative study themes,^33^ including opinions about firearm access assessment appropriateness and barriers to reporting access. Specific questions were pilot tested among qualitative researchers, clinicians, firearm owners, and people with suicidality experience. Guides began with rapport-building questions, followed by probes about experiences, opinions, and suggestions (eMethods 2 in the Supplement). Interviewers were purposely blinded to patient’s firearm access and suicidal thoughts responses to minimize implicit biases^44^ and to protect confidentiality. Interviews were audio recorded and professionally transcribed. Participants received a $50 incentive for participation.

### Statistical Analysis

Electronic health records data were used to describe patient participant characteristics and self-report used to classify clinician demographic characteristics including race and ethnicity. Study interviewers (J.R., E.K., and L.S.) coded transcripts using software (Atlas.ti, version 8; Scientific Software Development) using both directive (deductive) and conventional (inductive) content analysis.^45^ Two staff members (J.R. and L.S.) independently coded each transcript with iterative comparison and discrepancy resolution during weekly meetings. Patient interviews were analyzed first, and codes were organized into thematic networks^46^ to facilitate discussions. Clinician interviews were then coded and triangulation methods used to intersect clinician themes with patient themes (eMethods 3 in the Supplement).^47^

## Results

### Participant Characteristics

Thirty-six patients (mean [SD] age, 47.3 [17.9] years; 19 [53%] were male; 17 (47%] were female; 27 [75%] were White; 3 [8%] were Black; and 1 [3%] was Hispanic) were interviewed from November 18, 2019, to February 10, 2020, of 76 sampled (5 had nonworking telephone numbers, 9 refused, and 26 did not respond). Participants included 17 women and 19 men aged 19 to 87 years; at the time of sampling, 16 had reported firearm access and 15 had reported thoughts of self-harm (Table 1). Patient interviews lasted a mean (SD) of 19.5 (5.9) minutes (range, 11.0-33.8 minutes).

**Table 1.** Characteristics of Interviewed Patients and Clinicians

Characteristic	No. (%)	
	Patients	Clinicians
Total No.	36	30
Sex		
Women	17 (47)	24 (80)
Men	19 (53)	6 (20)
Age, mean (SD), y	47.3 (17.9)	44.3 (12.1)
Age category, y		
19-29	8 (22)	1 (3)
30-49	11 (31)	20 (67)
50-64	9 (25)	6 (20)
≥65	8 (22)	3 (10)
Race and ethnicity^a^		
American Indian or Alaska Native	0	1 (3)
Asian or Pacific Islander	3 (8)	5 (17)
Black	3 (8)	2 (7)
Latinx or Hispanic	1 (3)	4 (13)
Unknown	2 (6)	0
White	27 (75)	18 (60)
Firearm access^b^		
No	9 (25)	NA
Yes	16 (44)	NA
Not answered	11 (31)	NA
PHQ-9 Score, mean (SD)^c^^,^^d^	10.1 (8.1)	NA
Depressive symptoms^c^		
None/minimal (0-4)	12 (34)	NA
Mild (5-9)	6 (17)	NA
Moderate (10-14)	7 (20)	NA
Moderately severe (15-19)	2 (6)	NA
Severe (20-27)	8 (23)	NA
Frequency of suicidal thoughts (PHQ-9 Q9)^e^		
Never (0)	21 (58)	NA
Several days (1)	10 (28)	NA
More than half the days (2)	4 (11)	NA
Nearly every day (3)	1 (3)	NA

Abbreviations: NA, not applicable; PHQ-9, Patient Health Questionnaire.

^a^ Race and ethnicity were classified according to participant self-report from designations in electronic health records.

^b^ Patient response recorded on the Kaiser Permanente Washington (KPWA) mental health monitoring questionnaire used for criterion sampling within the 2 weeks prior to the recruitment initiation. The question was “Do you have access to guns? yes/no.”

^c^ Patient responses recorded on the KPWA mental health monitoring questionnaire used for criterion sampling within the 2 weeks prior to the recruitment initiation.

^d^ PHQ-9 used to measure depressive symptom severity.

^e^ In response to question 9 (Q9), “Thoughts that you would be better off dead, or of hurting yourself” (prior 2 weeks).

Thirty clinicians (mean [SD] age, 44.3 [12.1] years; 24 [80%] were female; 6 [20.0%] were male; 18 [60%] were White; 5 [17%] were Asian or Pacific Islander; and 4 [13%] were Latinx or Hispanic) were interviewed from July 7, 2020, to October 8, 2020, of 51 sampled (3 refused, 18 did not respond). Participants included 24 women and 6 men, 25 LICSWs and 5 RNs. Among LICSWs, 12 worked in both primary and urgent care, 13 in primary care only. Clinician interviews lasted a mean (SD) of 23.9 (5.9) minutes (range, 13.0-42.4 minutes).

### Organizing Themes

Key organizing themes derived from content analyses included (1) perceived value of standardized questions about firearm access, (2) challenges of asking and answering, and (3) considerations for practice improvement. Clinician interview themes did not diverge and often converged and/or complemented patient interviews (Figure). Quotations attribution reporting are as follows: A and B indicate patients in the first and second wave of interviews (respectively), and P indicates clinicians.

**Figure.**
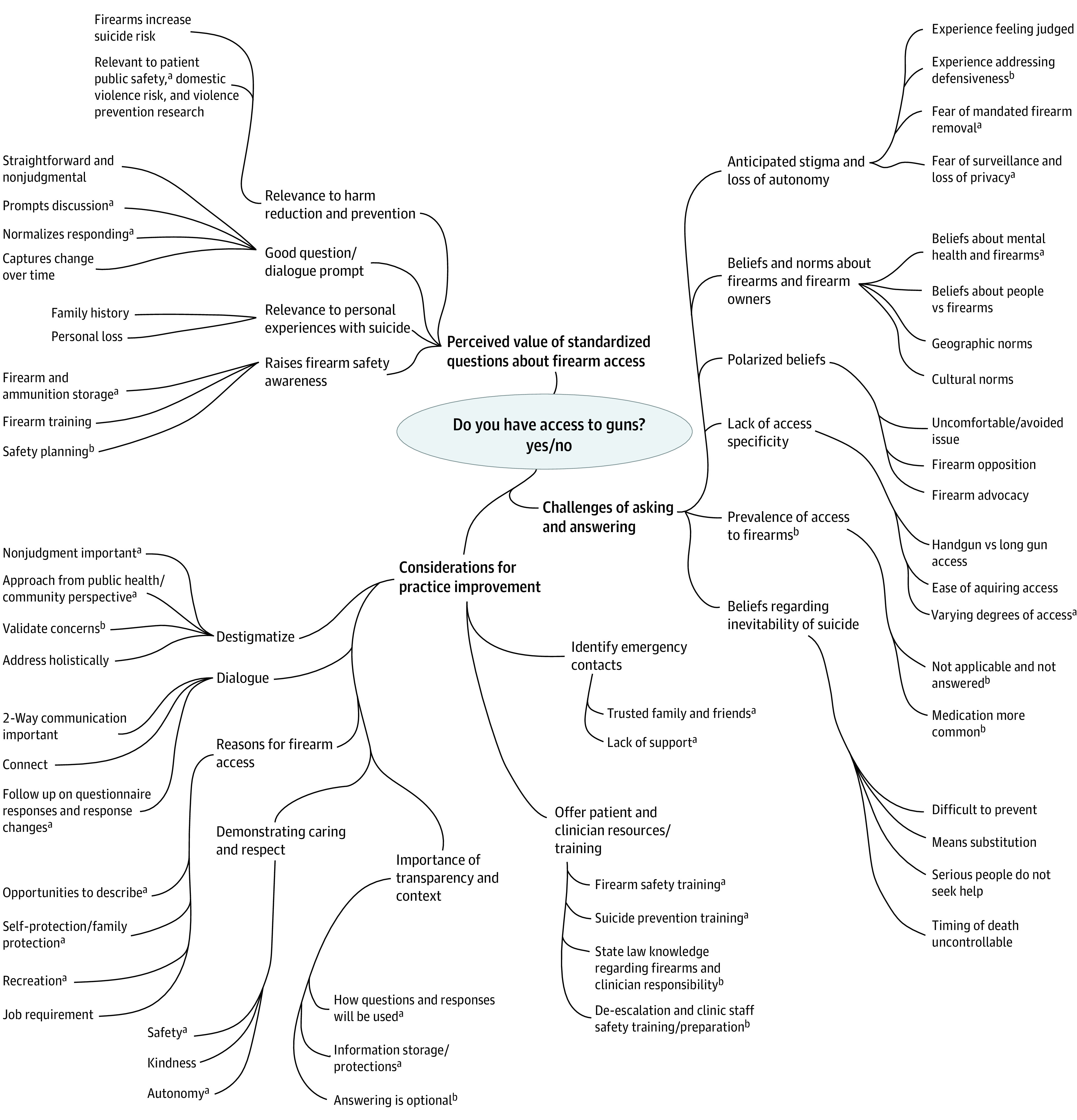
Thematic Network ^a^Convergent theme (patients and clinicians). ^b^Complementary theme (clinicians).

### Perceived Value of Standardized Questions About Firearm Access

Patients and clinicians were generally positive about firearm access questions. Patients described their relevance to harm reduction/prevention and suicide loss, and value as a dialogue prompt and for raising firearm safety awareness (Table 2). Regarding harm reduction, one said, “I completely see the relevancy of asking about firearms… whether it's someone who is struggling with depression, possibly suicidal thoughts, or someone who is struggling with feeling like they want to harm others. Asking about access to firearms seems like a very justifiable and relevant question (A010).” Some patients also described how firearms are highly lethal and relevant to personal and public safety, and domestic violence risk. Clinician interviews converged with this point; one said, “It's a public safety… public health issue, if there are kids in the house or other people who might accidentally use it to hurt themselves or somebody else. Just safety in general, even if it's not related to their own self harm (P14).”

**Table 2.** Perceived Value of Standardized Questions About Firearm Access

Theme	Example quotation^a^
**Relevance to harm reduction and prevention**	
Firearms increase suicide risk	A001: I grew up around them [firearms]… I also totally recognize that access to firearms, when you’re dealing with severe chronic depression or some other condition, really significantly increases risk of suicide. So it's a question that needs to be there [mental health questionnaire]. A024: Guns literally have the ability to have life or [cause] death. In some way it should be talked about in a health care appointment, like a doctor's office, because if someone is not in the right mental place, a gun could be the worst thing to have around.
Relevant to: patient and public safety, domestic violence risk, and violence prevention research^b^	A030: They [clinicians] want to make sure their patients are safe and they have good mental stability and health. The last thing you need is someone who's mentally unstable grabbing a firearm of any kind, and deciding to end somebody's world, either their world or someone's else's world. P14: I will also say it's a public safety issue in general, public health issue, if there are kids in the house or other people who might access it and accidentally use it to hurt themselves or somebody else. Just safety in general, even if it's not related to their own self harm. B037: We just had a mass shooting in downtown Seattle last night, so clearly talking about guns unfortunately probably needs to be done. B019: Increased access to weapons and guns can lead to a higher risk of domestic violence and suicide. B012: We need to have research on this, public health research on guns, because maybe that will help us have better control… I was just looking in the paper this morning, there was 3 different mass shootings in this country yesterday.
**Good question/dialogue prompt**	
Straightforward nonjudgmental	B012: It seems pretty straightforward and nonjudgmental, in my memory… I can't think of a better way to phrase it. A032: It's kind of blunt, but it's a good question… I'm not really sure how you could better ask that question.
Prompts discussion^b^	B025: I think you have to start with the paperwork so maybe that physician can get just a thought, or who's ever reading this information - hey, we have a person here that's trying to reach out. Then the next step is maybe one-on-one. P15: I mean, if you don't ask the question, you won't know (laughs). So you've got to be willing. And like I said, there have been other staff members who are a little nervous about asking. So I think it's good that it's on the questionnaire as a door opener. P14: That's the opener for me. ‘Hey, I'm looking at this form that you filled out and I noticed that you checked a box, or I noticed that you didn't check that box – was that intentional?’ It is really helpful to have that lead-in.
Normalizes responding^b^	A001: I answered yes. It didn't strike me one way or another as weird or anything; it seems like something that should be pretty normal. P23: I think that question on this form is so helpful. It just legitimizes the question and normalizes it.
Captures change over time	B009: I had firearms and they were under very strictly controlled access, we were very cautious about it. And then we decided, I really didn’t need them or want them, and they’re gone. So I had a string of “yes, yes, yes, no” on the questionnaire. That was interesting.
**Relevance to personal experiences with suicide**	
Family history Personal loss	B037: My father happened to commit suicide by shooting himself… So if you know that, and someone’s coming to see you and they’re depressed and they have access to guns, initially you might have to explore a little bit further… B026: For my situation with my daughter, her boyfriend did not lock the gun up, so she had complete access to it, and took it [died by firearm suicide]… when you’re in that mode, in that dark place, there’s really nothing. You think you’re really doing the world a favor.
**Raises firearm safety awareness**	
Firearm and ammunition storage^b^	B039: Most people don't seem to lock their guns up and that's where a lot of people are being killed because somebody didn't know the gun was loaded or the children have access to a weapon because it's being stored in a closet on a shelf somewhere…That's a good question to at least raise the awareness of people that don't seem to have knowledge of what firearm safety means. P16: Generally speaking, if someone acknowledges that they have access to firearms, then we'll have a discussion about what they use to keep those items safe from themselves, from family members, who's in the household, what is their household situation like.
Firearm training	A033: I think we should ask too, when they're asking do you have access to a gun - do you have training in that gun? What is that gun primarily used for? Self-defense, home security? Do you have a profession that requires you to carry a gun?
Safety planning^c^	P12: It helps me as a provider know whether I need to do any safety planning around firearms if they are suicidal. P17: It helps me to determine whether I need to do further assessment. It helps me determine whether this patient could benefit from a crisis response plan. Hospitalization could be a consideration if they're unsafe. It helps me create a treatment plan for them.

^a^ Interviewees were identified as follows: A = patients in the first wave of interviews, B = patients in the second wave of interviews, P = health care clinicians. The number following each letter indicates participant number. Grammatic edits noted in brackets were added to clarify intended meaning.

^b^ Analysis triangulation between patient and clinician interviews identified convergent theme.

^c^ Analysis triangulation between patient and clinician interviews identified complementary theme.

Patients also highlighted how the standardized question was “straightforward and nonjudgmental,” normalized the question, prompted discussion, and captured change over time. Similarly, clinicians valued the firearm question as a conversation prompt. As one described, “Today I had a patient make a comment about the firearm question, and I said ‘well, let me give you some [information]’– it opens up dialogue; an opportunity to talk to patients and provide education about why we ask it (P18).”

Several patients described how the question was relevant to personal experiences with suicide loss and how the question raises firearm safety awareness. Clinician interviews extended this point by describing how the firearm question helped engage patients in collaborative safety planning. One said, “I always look at how they've responded to the question and then maybe use that as a jumping off point to talk about lethal means removal and what steps they could take to make the home environment safe (P20).”

### Challenges of Asking and Answering

Patients described various challenges associated with standard questions about firearm access, including anticipated stigma or loss of autonomy, beliefs and norms about firearms and firearm owners, polarized beliefs, lack of access specificity, prevalence of access, and beliefs regarding suicide inevitability (Table 3). First, firearm owners commonly described experiences feeling judged and concerns about mandated firearm removal, surveillance, and/or loss of privacy. Clinicians also described observing similar patient experiences in the context of nonresponse (often) or active refusal to answer the question (less common): “There's people who said that they won't answer, they have concerns about their rights or having that in their record. So there's been times where people are proactively defensive about their gun ownership and their legal rights, and protecting themselves and what they say… there's that handful of times where people say, ‘I'm not answering that question’ (P10).”

**Table 3.** Challenges of Asking and Answering Standard Firearm Access Questions

Theme	Example quotation^a^
**Anticipated stigma and loss of autonomy**	
Experience feeling judged	A019: It [reporting firearm access] might affect my treatment; I might be looked at differently. I've dealt with a lot of judgment and ridicule in my health care for other reasons, and I always feel like that [firearm access] might be one of them, and I don't feel like that's fair. B024: I've experienced with my mental health problems there are many situations [when] I just feel people are against me or trying to get information out of me, to turn it on me in a bad way… Especially in our society, because it's so taboo [firearm ownership], it's a nervous thing to talk about.
Experience addressing defensiveness^b^	P16: There's some stigma with certain men that I've worked with, that it's absurd for me to even ask the question would they misuse their firearm. I get a scoff a lot of times if I'm saying hey, do you have a lock on your firearm? And they're always like, “I don't need a lock on my firearm” or whatever. P14: It's typically a male who has very strong opinions about their rights to own a gun and have a lot of language around being safe and being trained. I totally get it’s their right, but when the defensiveness pops up that can be hard, even if they aren’t at risk. If they are at risk [for suicide], that gets a lot more difficult.
Fear mandated firearm removal^c^	B036: I'm worried if I answer the questionnaire in a particular way that my ability to continue owning firearms will be taken from me. A024: I guess a lot of people could take it [firearm question] the wrong way, like you're trying to take my guns away. A033: What I don't want is what's been discussed in politics lately; having hospitals, therapists, counselors, any medical professionals turning over data on individuals so they can go ‘this person's mentally unhealthy,’ and going in and taking their guns away. P10: there's been a few times where people are proactively defensive about their gun ownership and their legal rights, protecting themselves and what they say. And making it clear that's what they're doing. Acknowledging concerns about putting themselves in a position to potentially lose their access. Not that we had even addressed that or it was necessarily a concern, but there's that handful of times where people have been filling that out and been like ‘I'm not answering that question.’
Fear of surveillance and loss of privacy^c^	A024: That document could be kept for a long time. A lot of people, they're worried about somebody tracking them down, they need that gun for their safety. Now they're worried about the fact that they just gave formal documentation to a doctor that they have a firearm and now more people know about it and they'd be really scared. B012: I don't want our society to be a surveillance society, that concerns me... I want individuals to be protected, but when it becomes an overarching thing that everyone in the country must declare whether they're a gun owner or not, I wouldn't want to necessarily go there either. P20: People are afraid we’re somehow tracking gun owners, and that we’re going to initiate action against them or come to their home and recover their guns if they’ve answered questions a certain way on the questionnaire.
**Beliefs and norms about firearms and firearm owners**	
Beliefs about mental health and firearms^c^	A033: That's been brought up–people who have mental health issues shouldn't own guns. I'm a military veteran and I have the right to my gun and yes, I'm depressed because I just spent 5 years killing people in Afghanistan. Just because you have a mental health issue doesn't mean you're a danger or a threat, but a lot of people are looking at it that way. P12: One patient that did disclose that they had firearms and were having some suicidal thoughts I think was very nervous to disclose that because they thought that perhaps their civil liberties would be infringed upon by disclosing that, and they really wanted to continue to have access to their firearms.
Belief about people vs firearm	A009: If that gun is laying there on the table, perfectly harmless. But if an idiot picks up the gun, then you've got problems… It's the person that's behind the weapon that's dangerous.
Geographic norms	A001: I'm in Spokane. So people that live in Seattle in particular, they always freak out about it [firearms]. Meanwhile, over here in Spokane, it's just a normal part of life.
Cultural norms	B038: In my culture if you have gun at home, it's very scary and very dangerous and we're not allowed to have that or touch it or to play with it. It's like a bomb. It's very dangerous. If somebody has a gun at home, you better be careful.
**Polarized beliefs**	
Uncomfortable/avoided issue	A001: It's always a little bit weird because guns are a really politicized topic in America, obviously. A019: I feel like it's a very political issue now, and so I really try to avoid the topic.
Firearm opposition	A033: In health care, in the workplace if you mention you have a gun a lot of people get upset nowadays; they don't want anybody to have them. Unless you're a trained professional and even then, those are being questioned.
Firearm advocacy	B023: I self-identify as a gun nut, but watch out for the gun nuts, they're wily. Certain subsets of American culture have different attitudes toward guns, ranging from positively dangerous to just positive or negative. There's a church that believes the AR-15 is the rod that Jesus is going to rule with when he comes back?!
**Lack of access specificity**	
Handgun vs long gun access	A033: Just because you have access to a gun doesn't mean you've got a pistol… if you're going to shoot yourself, you're probably going to use a handgun, because trying to do that with a long-barreled rifle usually doesn't work.
Ease of acquiring access	A014: People who know firearms will tell you the same - it is easy to get a hold of firearms. I'm trying to best answer [firearm question] – so when it comes to talking about firearms in a health care setting, oh man, it's kind of difficult.
Varying degrees of access^c^	B023: Just because they're in a storage locker in a place that nobody knows about doesn't mean I don't have access, I do. It's just they're not in my face day to day. P42: it might be that there's a gun in the home but the person doesn't really have direct access to it, or they don't know where the bullets are, all kinds of various bits and pieces of differing situations. P25: I feel like it has to be clarified more, like what do you mean do you have access? Do you have access as in this gun is unlocked and you can get access to it? Or is it just at your house? There has to be follow-up questions or it has to be more specific, than just do you have access to a firearm.
**Prevalence of access to firearms**^b^	
Medications more common^b^	P16: Medications used for overdose [is] my more typical intervention. Firearms do not come up as often as I would think.
Not applicable and not answered^b^	P14: If I notice in the moment that it wasn't circled, I will ask, and they almost always say oh, I just didn't see it, or I didn't feel like that applied to me.
**Beliefs regarding suicide inevitability**	
Difficult to prevent	B021: Let's be honest - if somebody really wants to do something [die by suicide], they're going to do it. Or they're going to try and do it, regardless of whether you say something to them or not.
Means substitution	A032: There's so many other things that you could do, so it's like yeah, I don't have access to guns, but I have access to lots of other options.
Serious people don’t seek help	A015: The suicidal people that are serious about it don't tell everybody they're having an issue, it just kinda sorta happens and then boom, they're gone.
Timing of death uncontrollable	A009: You don't take your life… When the Good Lord says come home, you're going home. I don't care if that gun is there or not.

^a^ Interviewees were identified as follows: A = patients in the first wave of interviews, B = patients in the second wave of interviews, P = health care clinicians. The number following each letter indicates participant number. Grammatic edits noted in brackets were added to clarify intended meaning.

^b^ Analysis triangulation between patient and clinician interviews identified complementary theme.

^c^ Analysis triangulation between patient and clinician interviews identified convergent theme.

Patients also described awareness of beliefs and norms about firearms and firearm owners, including the belief that people experiencing mental health issues should not have firearm access. Patients also described how polarized opinions about firearm ownership could make firearm access a difficult and uncomfortable discussion topic. One said, “Obviously the long-term goal would be harm reduction, but how do you achieve that without wading into some really fraught political waters? (A001)”

Some patients described how access can mean different things, for example how access to a handgun vs long gun may be more relevant for suicide risk, how purchasing a firearm is often easy, and how different storage solutions may be helpful for limiting but not totally precluding access. One said “Semantics are important. I know what my mental distress is, I know what my problems are, so when I start having problems, I gather everything up, lock everything away. Things are secured normally, but then away-away, offsite, not where I live and work. That's usually my next step. But technically I still have access (B023).” Some clinicians also described how prevalence of access to firearms impacted their perception of the salience of assessing firearm access for suicide prevention.

A few patients also described beliefs about the inevitability and difficulty of preventing suicide, including how people who are serious about suicide will not seek help, how the timing of our death is outside of our control, and how there are many ways to die by suicide. One described, “It's like yeah, I don't have access to guns, but you have access to lots of other options (A032).” Clinicians similarly described how addressing access to other lethal means is important. One said, “It's absolutely helpful to know they may have access to firearms but there are other lethal means we need to know about…an obvious example is Oxy, opioids (P40).” A few clinicians additionally described time limitations as a reason why asking about firearms is difficult. One said, “If a patient has suicidal ideation and has a gun, I'm going to take the time I need to have that conversation, but always thinking about the context of how long will this conversation take (P18).”

### Considerations for Practice Improvement

Patients offered suggestions for destigmatizing conversations, opening dialogue, acknowledging reasons for firearm access, demonstrating care and respect, creating transparency, identifying emergency contacts, and offering patient/clinician–facing resources (Table 4). First, patients described how to destigmatize firearm access by emphasizing nonjudgment, approaching from a public health perspective, and discussing firearm access as a holistic part of health care. They also emphasized the importance of dialogue—2-way communication, connection, and follow-up on response to the firearm question. Clinician interviews extended this point; one said, “I think it's [firearm question] a good starting point, but I do a verbal follow up and re-ask if they have access to firearms or lethal means, even if they answer no or skip (P16).”

**Table 4.** Considerations for Practice Improvement

Theme	Example quotation^a^
**Destigmatize**	
Nonjudgment important	B023: The patient needs to feel like they're in a safe, judgment-free environment where they can speak freely and not have it be a moral or quantitative value judgment leveled at them. B024: I think [patients need] additional reassurance that nobody's out to get them or judge them based off these questions that they're answering.
Approach from public health/community perspective^b^	A001: Especially for men, finding a way to destigmatize that conversation... I don't know what the stats are like for the trans community which I'm a part of…finding some way to tailor that message to people so they [firearm owners] don't feel called out for having that discussion. A011: If there is an alignment between questionnaire information and the ability to help others - people might wonder why you're asking, but if it could help somebody and save a life, if that was the premise, that would be I think enticing. P14: Especially the times people have been [said] ‘why are you asking this?’ I will also say it's a public safety issue in general; a public health issue.
Validate concerns^c^	P5: Just being calm, normalizing the question, being patient, letting people voice concerns and validating those concerns when they are expressed.
Address holistically	B037: In general, talking about firearm safety is probably best done before somebody's suicidal. So maybe that's something that you help families with, as a holistic thing, talking to kids about it too. But the conversation needs to start long before someone's suicidal.
**Dialogue**	
2-Way communication important	B025: It's very private, it's probably hard for some people to express. They might not think it's any of their business to ask questions [about firearms]…I'm sure some people take offense, but how else are you going to find out? I think you have to do it that way; start and hopefully get a good relationship with your patients so that your communication is working both ways.
Connect	B026: Doctors are very busy and it's hard for them, because they have a 10 or 15 minute schedule with the patient. But I think it's important to just take a breath, sit down with them. I just think it's important, because there needs to be a connection.
Follow-up on questionnaire responses and response changes^b^	A033: The paper gives you a starting point, but it's up to the individual [provider] to look at it and go I think they are safe, I think they are a threat… The providers need to follow up a little bit, ask some questions. If they've been in a dozen times, they should know the answers at that point. And if the person changes something, then they need to look at it again. P20: I always look at how they've responded to the [firearm] question and then use that as a jumping off point to talk about lethal means removal and what steps they could take to make the home environment safe.
**Varied reasons for firearm access**	
Opportunities to describe^b^	B024: I just want to try to be a really good citizen, but I can understand how owning a gun might not look like that on paper until you explain yourself. Like no, I have it legally, etc. A010: If the patient checked “yes.” Maybe an optional, more specific question – what is your access? “I own a gun” or “My roommate has a gun” or “There's a gun shop right next door to my apartment complex.”… Maybe open-ended – is there any further information you would like to provide about your access to firearms? Which would open it up to the patient to provide more specific information about what their access is and even open up the option to discuss how they feel or what they think about the fact that they have access to firearms. P8: Sometimes you will have patients who want to describe more about their relationship with carrying and using it, having a weapon. I just have a huge variety of patients with that question.
Self-protection/family protection^b^	A030: I have a firearm for protection because before everyone in my family moved in with me, it was just me and my husband at home, and he works nights. I have a daughter who always has the wrong kind of characters hanging around her, so for us to feel safe, making sure I had something at home to protect myself [so] if anything really bad was to happen, I wouldn't be helpless. A014: The doctor asked me why do I have firearms and I told her I've been a victim many times, that's why I have it, just as a last resort. I don't want to be a victim anymore. A001: I am a rural queer, I live out in the country where guns are a part of our culture, but it's also a safety issue for us. A019: For me it's about protection for my home – for me. It [firearm] was inherited from my father. It's something that I choose to keep, to protect my home. I'm not the suicidal tendency person. Yes, I have depression, yes, I am bipolar, but I've never been able to go down that road [suicide]. So having that discussion I think reassured him [the doctor] that there's another reason why I have a firearm. P8: I've had multiple patients describe that they have some limitations in their mobility, or they're a single woman; they've described feeling unsafe, so feel the need to carry. P20: [One patient] mentioned that he’s a Black man, and feels that his family, his life has the potential to be under siege in a way that other people would not perceive their lives to be.
Recreation^b^	A022: I don't have one [a firearm] in my house, but if I wanted to use one, to go release some tension out in the woods, whatever, I got a friend's dad that has them and we're very careful. We shoot at a target only. P25: A patient had a gun from the 1970s that they used for hunting, that she hasn't used in 30 years… She repeated to me that it was a family gun they used when they were little - it was sentimental value. You really have to talk to them, because it's not just a straightforward yes or no answer.
Job requirement	A015: I am in law enforcement, so I have guns at my disposal.
**Demonstrating caring and respect**	
Safety^b^	B023: I think address it from the standpoint of trying to improve the patient's own safety culture… I work in construction, so safety culture is a big deal. There's not as heavy a safety culture emphasis in the broader gun culture, so if the health care provider could fill that gap a little bit, for some patients it may be a little more palatable. A024: We're thinking about you and trying to keep you safe and trying to keep you in this world because there's people that love you. P28: Some people refuse to answer it, but I think it elevates the conversation… that's good because out of that comes ‘there's people trying to take guns away…’ And I'm like, ‘what if we just took that off the table, and why I'm bringing this up is I'm concerned about your safety.’ So, when we get to that point, we can have a really good conversation about safety and firearms and if there is a need for lethal means removal.
Kindness	A033: If you talk to them as a person and go hey, we want to help, as opposed to… we're medical professionals, we're going to do this. You can't cram healing down anybody's throat… Treat them with kindness and respect, and that gets more out of people than just about anything else I can think of.
Autonomy^b^	B036: Even if they find it a risk that I own those things [firearms]. I guess in one sentence, help people understand they do have their own authority to make their own choices about what they own and what they have... P10: It helps to validate that because when people are a little more suspicious, that helps align like “I'm not here to try to take away anyone's rights or freedom.” P5: I own a firearm, I've handled firearms, I've shot firearms. I am comfortable for the most part with a firearm... but I typically don't bring my own experience. The few times I have, it's really been more – I've referenced the value of having the right to bear arms and that no one wants to take away your firearms or your guns permanently.
**Importance of transparency and context**	
How question responses will be used^b^	A015: There wasn't any explanation on why you were filling the form out… So maybe a little more explanation would be helpful. A032: My concern would be, what does it imply? What would change if I had access to guns vs not? Who would care, and respond? P4: I would explain why I was asking that question and how it pertains to us figuring out in what ways can we keep you safe. I felt like that definitely helped patients feel a little more comfortable when I would ask that question. P37: I think the strategy is to let them know this is not about us trying to control your guns. It's really to make sure that should they have an emotional swing, it's always nice to know the safety parameters of the gun in ways to help the situation be as safe as possible.
Information storage/protections^b^	B009: It might be helpful for some people to have a better idea of what might be done with the information they provide. People can be pretty paranoid about what might happen with the information. P10: When people have had concerns, I reiterate their protections within a clinical setting, regarding confidentiality and also the limits to confidentiality.
Answering is optional^c^	P40: People who have very strong opinions about their right to bear arms get really suspicious. I tell them if you don't want to answer, you don't have to answer. There's no requirement here. This is for your health. So I have had some people say ‘great, I don't want to answer,’ and then by the time we're done working together that day, they'll be like ‘oh, by the way, yeah, I do have a gun, but I have it locked away and the bullets are here” and bla bla bla bla. So it helps to reduce their defensiveness.
**Identify emergency contacts**	
Trusted family and friends^b^	A015: Somebody else has to intervene, but it has to be somebody that hypothetically I trust, that aren't going to take my guns and never give them back and put me in a mental hospital, because then that will make me want to get my guns. So maybe the doctor could inquire… maybe say hey, is there somebody with you or somebody close where we could have a more in-depth conversation about what you're going through? P5: Shore up family contacts and emergency contacts or people who are okay to contact in case of crises or thoughts of suicide, particularly if we can't reach the patient or if there's a concern and something to that effect.
Lack of support^b^	B023: I'm an example of this – not everybody has a friend or family member that they can say ‘hey, can you hold onto my firearms while I'm dealing with some stuff?’ In some families, some people just tell you to suck it up, which is not necessarily good mental health advice. P12: I sometimes fear that they [patients] won't have anyone that they feel like they can give it [firearm] to temporarily, or they don't have a safe or a safe place to store it, or they potentially don't want to do that. Although I think most people understand that if they're talking to me, they're wanting some kind of help, so I think most people would be willing to try to problem solve.
**Offer patient and clinician resources/trainings**	
Firearm safety training^b^	A001: There are organizations out there that are not the NRA that can teach firearm safety and there are organizations out there that will teach firearm safety especially to queer, LGBTQ people. I think if you had some brochures or something like that for people, that would be awesome. P18: I would like to see something more concrete in what we offer for firearm safety and firearm training.
Suicide prevention training^b^	B009: I'm in the Department of Defense and we have annual suicide prevention training. Of course, it talks about access to firearms and that sort of thing as a particular risk for people in DOD and it's a really good course. Making something like that available to people who may need it… P14: I wonder if we can bulk up comfort outside of the mental health department. As a system we are engaging in this and asking these questions [re suicidal thoughts and firearms], and lots of providers aren't and saying can you get the social worker? Which is fine, but also increasing comfort overall would be cool.
State law knowledge regarding firearms and clinician responsibility^c^	P21: I do think it would be helpful to have a better understanding of state law around firearm safety when it comes to suicide and mental health issues. We have so many providers, me included, who moved here from out of state and did their training out of state, and those laws really change when you go from one place to another. P12: I feel liable and want to make sure that I'm following the law as well making sure that the patient is safe…I don't know a lot about firearms and I don't know if that limits me in any way in those discussions. Perhaps it does. There may be safety measures or things that I'm completely unaware of, just because I've never been around them or used them personally.
De-escalation and clinic staff safety training/preparation^c^	P25: I had an 80-year-old patient who said she had a gun in her purse and that she was planning to use it… So I was in this room and I didn't know if she had a gun, but when I asked her, she was crying. I was like, we have to know if you have a gun. She was like no I don't, I just said it because I was frustrated. That needs to be addressed; the safety of the clinic. P21: We have had a couple of instances where patients make violent threats on the clinic, and I think those caught everybody by surprise…So I think there's maybe some work to be done on the planning and preparation side for care teams.

^a^ Interviewees were identified as follows: A = patients in the first wave of interviews, B = patients in the second wave of interviews, P = health care clinicians. The number following each letter indicates participant number. Grammatic edits noted in brackets were added to clarify intended meaning.

^b^ Analysis triangulation between patient and clinician interviews identified convergent theme.

^c^ Analysis triangulation between patient and clinician interviews identified complementary theme.

Patients routinely described important reasons for their firearm access, including self/family protection, recreation, and professional requirements. Clinician interviews extended this point; one said, “Some people will say it’s an heirloom rifle from my great-grandfather, and one person said to me, ‘I have a sacred duty to protect my family.’ You’re wanting to get to the underlying reason why it’s important for them to have the gun, to see where you might go from there (P20).”

Patients also described the importance of transparency, autonomy and context—understanding the purpose of the firearm access question. Patients and clinicians suggested discussing how patients’ responses will be used and protected. Clinicians suggested that emphasizing that responding is optional can help address defensiveness. One patient said, “There's a lot of concern… that the government is going to take [firearms] away, whether or not that's true. Transparency and being open will have a much better outcome than not being upfront about why you need the information (B029).”

Finally, some respondents emphasized the importance of identifying emergency contacts when possible, as well as firearm safety and suicide prevention training opportunities, especially for clinicians with less experience or comfort with these topics. Several clinicians additionally emphasized a need for information about state laws applicable to firearm safety, and how to handle rare but serious instances when they encounter patients who are stating intent to harm themselves or others.

## Discussion

This qualitative study purposefully elicited multidimensional experiences from patients who had recently answered and clinicians who had recently used a standard question about firearm access to support care delivery–a clinical practice that remains uncommon, despite potential benefits for suicide prevention.^20,48^ Their varied perspectives often converged and provided vital information for understanding how to improve this practice. Most respondents perceived the standard firearm question as valuable for opening dialogue about suicide risk and harm reduction and also described important challenges with eliciting this information. Respondents’ practice improvement suggestions emphasized the importance of nonjudgmental acknowledgment of patients’ varied reasons for firearm access.

These findings, in combination with prior qualitative stakeholder-informed research,^30,32,35,36,37,38,49,50,51,52,53,54,55,56^ provide a road map for addressing barriers clinical teams may experience with firearm access assessment. This study underscored the need to communicate that the intended purpose of routine questions about firearm access is to support conversations about suicide prevention, not to limit patient autonomy.^30,31,32,33^ This study extended prior qualitative research on suicide risk identification,^33,57^ with suggestions on how to use standardized questions to normalize, destigmatize, and encourage dialogue about firearm safety. Specifically, respondents commonly described the importance of opportunities to discuss the reasons for firearm access, which often included self-protection, consistent with national surveys.^4,6,58^ Prior qualitative research has connected experiences with violence to possession of firearms for self-protection.^35,36,59^ Therefore, trauma-informed approaches^60^ to addressing firearm access may be particularly impactful for patients, especially in combination with education about our natural human tendencies to consistently overestimate our own ability to make rational decisions while experiencing intense emotions (ie, “hot states”), such as anger and pain.^61^

This research has 3 main clinical implications. First, respondents understood and valued the prevention potential of the firearm access question. This finding aligns with our cross-sectional research demonstrating most patients receiving mental health care will answer this question,^40^ and prior research indicating that people are generally receptive to firearm injury/suicide prevention practices.^30,38,49,50,51,52,56,62,63^ Second, respondents described a nuanced understanding of challenges that may drive under-reporting access. Our recent retrospective cohort study found that over half the patients who died by firearm suicide during a 4-year period reported no firearm access in the year prior to death.^64^ While some suicide decedents likely acquired firearms after reporting no access, others likely chose not to disclose access.^64^ Third, respondents described how standard firearm access questions help normalize clinician-initiated dialogue to support patient safety and suicide prevention. Several prior studies have proposed that universal firearm injury prevention counselling may be more palatable than asking patients about access.^30,32,37,54^ The results of this study do not preclude universal counseling, but suggest a *both/and* rather than *either/or* approach could be useful (ie, standard questions + dialogue).^65^

### Limitations

This study has limitations. First, KPWA only uses the standardized firearm access question with adults receiving mental health care during primary care and mental health specialty encounters.^40^ Therefore, sampled respondents included those for whom disclosing firearm access may be most important; future research is needed to generalize findings to broader patient populations and increasingly common virtual care encounters.^66^ Second, KPWA serves primarily urban and suburban populations; experiences reporting firearm access may differ in rural regions where firearm access is more common.^51^ Third, Washington State has an Extreme Risk Protection Order (EPRO) process, allowing temporary firearm removal when individuals are legally determined to pose danger to themselves or others.^67,68^ EPRO awareness may have increased both patient apprehension of mandated firearm access removal and clinician desire for education about regulations. As states adopt EPROs, there will be increasing need to understand their impact.^69,70,71,72^ Lastly, the firearm access question KPWA uses is purposefully not specific about what access may mean (as respondents described). Additional research is needed to evaluate and improve questions designed for firearm suicide prevention.

## Conclusions

In this qualitative semistructured interview study with patients and clinicians, findings suggested standard firearm access questions can normalize and support dialogue. Transparency and context, combined with a trauma-informed approach to initiating dialogue about limiting firearm access, may be particularly helpful. Understanding functions (ie, purpose) firearms serve for patients may help clinicians discuss planning for those times when decision-making abilities are impaired. Future development of firearm suicide prevention strategies and resources should include nonjudgmental acknowledgment of reasons for firearm access to support engagement in collaborative patient-centered dialogue about when and how to limit access to firearms to reduce risk of suicide.

## Notes

Supplement. **eMethods 1.** Additional Information Regarding Sampling and Data Management **eMethods 2.** Interview Guides **eMethods 3.** Additional Information Regarding the Data Triangulation Process
eReferences
