# Supplementary material for: Patient and Clinician Perspectives of a Standardized Question About Firearm Access to Support Suicide Prevention: A Qualitative Study
Source: JAMA Health Forum. 2022 Nov 23;3(11):e224252. doi: 10.1001/jamahealthforum.2022.4252 (PMC9685488; doi:10.1001/jamahealthforum.2022.4252)
Supplement: Supplement. — eMethods 1. Additional Information Regarding Sampling and Data Management eMethods 2. Interview Guides eMethods 3. Additional Information Regarding the Data Triangulation Process eReferences [file jamahealthforum-e224252-s001.pdf]

Supplemental Online Content

Richards JE, Kuo ES, Whiteside U, et al. Patient and clinician perspectives of a standardized question about firearm access to support suicide prevention: a qualitative study. *JAMA Health Forum.* 2022;3(11):e224252. doi:10.1001/jamahealthforum.2022.4252

eMethods 1. Additional information regarding sampling and data management

eMethods 2. Interview guides

eMethods 3. Additional information regarding the data triangulation process

eReferences

This supplemental material has been provided by the authors to give readers additional information about their work.

© 2022 Richards JE et al. *JAMA Health Forum*.

eMethods 1. ADDITIONAL INFORMATION REGARDING SAMPLING AND DATA MANAGEMENT

Below is a description of the process used to purposefully sample patient participants for interview participation, used in combination with data management strategies designed to support data collection quality and protect patient privacy**.**

Goals

- To recruit and adult patients according to a specific stratified sampling distribution (detailed below).[1]
- To minimize interviewer implicit bias,[2] known to be associated with suicidality[3 4] and firearm ownership,[5 6] during the recorded interview.
- To protect patient privacy by minimizing the use of patient-reported firearm access and suicidality reported on the mental health monitoring tool until study participants provide informed oral consent.

Step 1: Define sample using electronic health record data (study programmer)

A) Identify adult (Aged 18+) patients who received the mental health questionnaire (with firearm access question) within prior 2 weeks. If multiple tools were completed, pulled the most recent incidence.

Notes: Include only mental health monitoring tools associated with in-person encounters (i.e., virtual encounters rare at the time of this evaluation)—office visit, mental health visit, preventive care visit, or social work encounter to Family Medicine, Social Work or Mental Health Services, defined via screening dept specialty).

B) Use responses to firearm question (*Do you have access to guns? Yes/No)* to include a stratified distribution of target interviewees who answered “yes” (target 30%), “no” (target 30%), and who did not respond (target 30%).

Notes: Non-response was defined using methods previously developed to analyze whether and how patients responded to the standard firearm access question.[7]

C) Within each group (defined above), include approximately half of people who also reported some level of suicidal ideation using responses to the ninth question of the 9-item Patient Health Questionnaire (PHQ-9) regarding frequency in prior 2 weeks (*Thoughts that you would be better off dead or of hurting yourself in some way*) by selecting those who responded, *Several Days-1, More than half the days-2, or Nearly every day-3*.[8-11]

D) Exclude patients with prior year dementia diagnosis, patients under age 18 (at time of answering mental health monitoring tool), and patients who proactively opted out of contact for research participation.

Step 2: Create a recruitment sample (study programmer)

Assign a unique study ID to the first wave of sampled participants (target N=30). Populate REDCap tracking database accessible to the study interviewers that includes patient names and contact information (mailing address and phone numbers). Track age,

© 2022 Richards JE et al. *JAMA Health Forum.*

sex, race, ethnicity, and responses to mental health monitoring tool questions in a separate location accessible only to the study programmer (by assigned study ID).

Step 3: Adjust sampling targets (study programmer and project leader)
As participants were recruited for interview participation, study interviewers send the study programmer the study IDs of patients who had been interviewed. The study programmer populates spreadsheet to review in collaboration with the project leader and adjust sampling targets for subsequent recruitment (repeating Step 2).

Step 4: Prepare analytic dataset for qualitative analysis (study programmer and project leader/analyst)
Once the study interviewer/coders determine that thematic saturation has been reached, the programmer prepares an analytic dataset that includes the study ID, demographic characteristics and patient responses to the mental health monitoring questionnaire. The study PI (JR) uploads individual-level demographic, firearm access and suicidality information into ATLAS.ti to assist with qualitative analysis. In parallel, members of the study team (JR, LS) prepare an aggregated descriptive summary of participant characteristics for presentation.

© 2022 Richards JE et al. *JAMA Health Forum*.

eMethods 2. INTERVIEW GUIDES

Semi-Structured Patient Interview Guide

Introduction
Thank you for talking with me today. Before I start the recorder, I want to let you know I'm going to try not to use your name during the recorded part of the interview. If you avoid using names too it will help us protect your confidentiality.

Our conversation today will be about your experience in your provider's office filling out a questionnaire about your mental health that asks about depression, thoughts about self-harm, substance use and firearm access. We want to better understand healthcare's role in helping patients stay safe by finding new ways KP can communicate with patients about access to guns.

I don't know how you answered those questions and I'm most interested in your experience answering and less about how you actually answered. Does that make sense? I won't be asking you how you answered, but you are welcome to share that information with me.

Is it ok with you if I turn on the recorder now?
Do you remember recently filling out the mental health monitoring questionnaire (usually a green paper form) with questions about mood, alcohol and drug use, and access to firearms? It starts off, *over the past 2-weeks how often have you been bothered by any of the following symptoms? Little interest or pleasure in doing things; feeling down, depressed or hopeless…etc.*
[If no memory] It's ok if you don't remember completing it, we would like participants to have a memory of the question, but it's not required for the interview, so we'll keep going.
[Warm up question]

1. Tell me about your recent experience answering that mental health questionnaire? [Or if no memory, general experience answering questions about mental health?]

2. The last question on the mental health monitoring tool asks, Do you have access to guns? Yes/No. Can you tell me about your experience answering that question?
    - Tell me about your reaction to that question?
    - Tell me about concerns (if any) you had (or have) about the access to gun question?

3. In general, how do you feel about talking about guns in a healthcare context?

© 2022 Richards JE et al. *JAMA Health Forum.*

- What about non-healthcare settings, like at home with friends and family?

4. How do you think your provider uses patient answers about access to guns?
    - How about healthcare organizations more generally, like KP Washington?

5. Any suggestions or ideas for improving the way KP asks patients about firearm access?
    - In the context of mental health and suicide prevention specifically, any ideas on how KP could make it easier for patients to answer questions about firearm access?
    - What additional info might be helpful in this context (if any)? For example, would information about the connection between firearm access and suicide risk be helpful?
    - Would you prefer that info be provided as part of the questionnaire, part of a one-on-one conversation or some other way?

6. Have you ever talked with a healthcare provider about access to guns?
   [IF YES]: Can you tell me more about that experience? What was helpful or not helpful about how your health care providers (or members of their team) responded?
   [If NO] What about more generally about lethal means safety, like safe storage of prescription medications?

7. *Switching topics a bit, we are hoping to get your feedback about a new web-based resource we'll be giving patients. You may be able to answer based on personal experience but answering hypothetically is ok too.
    - When patients report frequent suicidal thoughts, providers will provide an anonymous online tool to help them make decisions about lethal means safety, like storing medications and firearms. What do you think providers could do to make this interaction feel safe and comfortable for the patient?
    - Based on your experience or hypothetically, how would you want to hear about this tool if you were thinking about suicide and had access to firearms?
    - How would you want your provider to start this conversation?
    - What would maybe make you more likely to try out the web-based tool?

[Wrap up question]

© 2022 Richards JE et al. *JAMA Health Forum*.

8. Is there anything else you would like to share that you think would be helpful or important to tell us?

******This question was designed to support a separate implementation evaluation of a web-based decision aid for reducing access to firearms (Lock2Live).*

© 2022 Richards JE et al. *JAMA Health Forum*.

Semi-Structured Clinician Interview guide

Introduction

Thank you for talking with me today. Our conversation today will be about helping patients stay safe by finding new ways KP can communicate with patients about access to firearms. We're curious how you currently use the information on patient-reported access to firearms and your thoughts about a new web-based decision aid designed to help patients at risk of suicide secure firearms and prescription medications. Before I start the recorder, I want to let you know I'm going to try not to use your name during the recorded part of the interview. If you avoid using names too it will help us protect your confidentiality.

Is it ok with you if I turn on the recorder now?

1. The last question on the Behavioral Health Monitoring tool asks patients "Do you have access to guns? Yes/No."
    a. What are your thoughts on asking patients about firearm access on self-administered questionnaires like these?
    b. What do you think patients think of this question?

2. How do you use information about firearm access generally?
    a. How about specifically when you are working with patients at risk of suicide, for example when patients indicate they are having suicidal thoughts on the PHQ-9 or have a history of suicide attempts?
    b. Can you give me an example (without disclosing patient names) of how you address firearm[s] when patients identified at risk of suicide indicate they have access to firearms?

3. In general, how comfortable are you talking about firearm safety with patients?
    a. How about specifically with patients at risk of suicide?
    b. How frequently do you address this topic (firearm safety in context of suicide risk)?
    c. What is your personal experience with firearms and how do you bring that experience into discussions with patients?

4. What barriers have you encountered when discussing or attempting to discuss firearm safety (e.g. safety storing firearms and ammunition) with patients?
    a. What about specifically in the context of suicide risk (e.g. temporarily storing firearms outside the home or other ways to help patients put time and distance between themselves and the firearm)?
    b. How about during virtual visits over the phone or by video?

© 2022 Richards JE et al. *JAMA Health Forum*.

5. On the flip side, what facilitators/strategies have helped you with addressing access to firearms with patients?
    a. What about specifically in the context of suicide risk?

6. *Switching topics a bit, we are hoping to get your feedback about a website that guides patients through a series of questions and concludes with personalized recommendations for safe storage of firearms. Clinicians and patients, including firearm owners and those with suicidal thoughts helped develop this web-based decision aid called Lock2Live. Providers can now enter a standard .dot phrase *.LOCK2LIVE* to add a short URL and a QR code for easy access to this resource in a crisis response plan or after-visit summary.
    a. What are your thoughts about this decision aid?
    b. We talked to patients about their preferences, some told us a simple link in a secure message would be fine, but others said they would be a lot more likely to try Lock2Live if their providers tried it with them first particularly in cases when they were feeling really depressed and suicidal. What are your thoughts about how providers could encourage their patients with firearm access and suicide risk to use Lock2Live?
    c. What tools or prompts might be useful for helping you introduce Lock2Live and encourage patients to try it? Would automatically populating the crisis response plan template with a URL and QR code helpful? What other ideas do you have?
    d. What kind of follow-up do you think might be helpful after the introduction of Lock2Live? Would some kind of prompt to check-in with patients at risk of suicide be helpful? Do you have tools you utilize now for that follow-up?

7. What additional tools or resources might be helpful for providers to address firearm safety with patients?

8. What other thoughts (if any) do you have about this topic you'd like to share?

**This question was designed to support a separate implementation evaluation of a web-based decision aid for reducing access to firearms (Lock2Live).*

We just have a few final demographic questions:

1. How would you describe your race/ethnicity? (tell me all that apply)
    a. Asian
    b. American Indian/Alaska Native

© 2022 Richards JE et al. *JAMA Health Forum*.

   c. Black or African American
   d. Hispanic or Latino
   e. Native Hawaiian or Other Pacific Islander
   f. White
   g. Other? _____________

2. What is your gender?
   a. Female
   b. Male
   c. Non-binary

3. What is your age (in years)? _____

© 2022 Richards JE et al. *JAMA Health Forum*.

eMethods 3. ADDITIONAL INFORMATION REGARDING THE DATA TRIANGULATION PROCESS

Below is a description of the purpose, methods and process developed to support the qualitative data triangulation approach used for this analysis.

Purpose:

To employ qualitative data triangulation methods to support analysis of *convergence*, *complementarity,* and *divergence* between two interview respondent groups.[13] We will explore *convergence* to enhance the validity of our findings, we will explore *complementarity* to support a multidimensional understanding of our research questions, and we will explore *divergences* to understand important differences in findings between respondent groups.

Triangulation Methods:

1. Data Sources: Separate thematic analyses of two respondent groups will be used for triangulation.
2. Multiple investigators: A small team of 4 researchers will work together to draft, refine and present findings to the full research team and project stakeholders.

Triangulation Processes:

Step 1: The lead researcher (JR) will make a preliminary assessment of how themes from the analysis of the clinician interviews converge, complement, or diverge from themes from the patient interviews. This investigator will also note which themes are present for one group of respondents but not the other (i.e. areas of "silence").

Step 2: The lead researcher will collaborate with a second researcher (LS) during weekly team meetings to review and further document areas of agreement in the meaning of the themes between respondent groups, partial agreement in meaning from different perspectives, and divergence in the meaning of themes between groups.

Step 3: Findings will be further refined in collaboration with two additional team members during bi-weekly team meetings—the third coder responsible for independently coding the clinician interviews (EK) and (a psychologist with experience treating suicidal patients (UW).

Step 4: The team responsible for triangulation assessment will present findings to the research team and stakeholders for further review and clarification.

© 2022 Richards JE et al. *JAMA Health Forum.*

eReferences

1. Patton MQ. Qualitative interviewing. Qualitative research and evaluation methods. Thousand Oaks, CA: Sage 2002:344-47.
2. Blair IV, Steiner JF, Havranek EP. Unconscious (implicit) bias and health disparities: where do we go from here? *Perm J* 2011;15(2):71-8. [published Online First: 2011/08/16]
3. Richards JE, Whiteside U, Ludman EJ, et al. Understanding Why Patients May Not Report Suicidal Ideation at a Health Care Visit Prior to a Suicide Attempt: A Qualitative Study. *Psychiatr Serv* 2019;70(1):40-45. doi: 10.1176/appi.ps.201800342 [published Online First: 2018/11/21]
4. Sheehan L, Oexle N, Armas SA, et al. Benefits and risks of suicide disclosure. *Soc Sci Med* 2019;223:16-23. doi: S0277-9536(19)30023-1 [pii]

10.1016/j.socscimed.2019.01.023 [published Online First: 2019/01/29]
5. Parent B. Physicians Asking Patients About Guns: Promoting Patient Safety, Respecting Patient Rights. *J Gen Intern Med* 2016;31(10):1242-5. doi: 10.1007/s11606-016-3694-2

10.1007/s11606-016-3694-2 [pii] [published Online First: 2016/05/22]
6. Guns Have Divided America. Here's What Happens with 245 People Try to Meet in the Middle. TIME, 2018, Vol 192 No 18.
7. Richards JE, Kuo E, Stewart C, et al. Self-reported Access to Firearms Among Patients Receiving Care for Mental Health and Substance Use. *JAMA Health Forum* 2021;2(8):e211973-e73. doi: 10.1001/jamahealthforum.2021.1973
8. Kroenke K, Spitzer RL, Williams JB. The PHQ-9: validity of a brief depression severity measure. *J Gen Intern Med* 2001;16(9):606-13. doi: jgi01114 [pii] [published Online First: 2001/09/15]
9. Louzon SA, Bossarte R, McCarthy JF, et al. Does Suicidal Ideation as Measured by the PHQ-9 Predict Suicide Among VA Patients? *Psychiatr Serv* 2016;67(5):517-22. doi: 10.1176/appi.ps.201500149 [published Online First: 2016/01/15]
10. Simon GE, Rutter CM, Peterson D, et al. Does response on the PHQ-9 Depression Questionnaire predict subsequent suicide attempt or suicide death? *Psychiatr Serv* 2013;64(12):1195-202. doi: 10.1176/appi.ps.201200587 [published Online First: 2013/09/17]
11. Simon GE, Coleman KJ, Rossom RC, et al. Risk of suicide attempt and suicide death following completion of the Patient Health Questionnaire depression module in community practice. *J Clin Psychiatry* 2016;77(2):221-7. doi: 10.4088/JCP.15m09776 [published Online First: 2016/03/02]
12. Tong A, Sainsbury P, Craig J. Consolidated criteria for reporting qualitative research (COREQ): a 32-item checklist for interviews and focus groups. *Int J Qual Health Care* 2007;19(6):349-57. doi: mzm042 [pii]

10.1093/intqhc/mzm042 [published Online First: 2007/09/18]
13. Farmer T, Robinson K, Elliott SJ, et al. Developing and implementing a triangulation protocol for qualitative health research. *Qual Health Res* 2006;16(3):377-94. doi: 10.1177/1049732305285708 [published Online First: 2006/02/02]

© 2022 Richards JE et al. *JAMA Health Forum.*
